# A tailored approach in lymph node-positive perihilar cholangiocarcinoma

**DOI:** 10.1007/s00423-021-02154-4

**Published:** 2021-06-01

**Authors:** Christian Benzing, Felix Krenzien, Alexa Mieg, Annika Wolfsberger, Andreas Andreou, Nora Nevermann, Uwe Pelzer, Uli Fehrenbach, Lena Marie Haiden, Robert Öllinger, Wenzel Schöning, Moritz Schmelzle, Johann Pratschke

**Affiliations:** 1 Department of Surgery, Campus Charité Mitte|Campus Virchow-Klinikum, Experimental Surgery and Regenerative Medicine, Charité – Universitätsmedizin Berlin, Augustenburger Platz 1, 13353 Berlin, Germany; 2 Department of Hematology, Oncology and Tumor Immunology, Charité – Universitätsmedizin Berlin, Berlin, Germany; 3 Department of Radiology, Charité - Universitätsmedizin Berlin, Berlin, Germany

**Keywords:** Lymph node positive perihilar cholangiocarcinoma, Major hepatectomy, Long-term survival, Postoperative complications

## Abstract

**Purpose:**

Extended right hepatectomy is associated with wide surgical margins in PHC and often favored for oncological considerations. However, it remains uncertain whether established surgical principles also apply to the subgroup of node-positive patients. The aim of the present study was to define a tailored surgical approach for patients with perihilar cholangiocarcinoma (PHC) and lymph node metastases.

**Methods:**

We reviewed the course of all consecutive patients undergoing major hepatectomy for PHC between 2005 and 2015 at the Department of Surgery, Charité – Universitätsmedizin Berlin.

**Results:**

Two hundred and thirty-one patients underwent major hepatectomy for PHC with 1-, 3-, and 5-year overall (OS) and disease-free survival (DFS) rates of 72%, 48%, and 36%, and 60%, 22%, and 12%, respectively. In lymph node-positive patients (*n* = 109, 47%), extended left hepatectomy was associated with improved OS and DFS, respectively, when compared to extended right hepatectomy (*p* = 0.008 and *p* = 0.003). Interestingly, OS and DFS did not differ between R0 and R1 resections in those patients (both *p* = ns). Patients undergoing extended left hepatectomy were more likely to receive adjuvant chemotherapy (*p* = 0.022). This is of note as adjuvant chemotherapy, besides grading (*p* = 0.041), was the only independent prognostic factor in node-positive patients (*p*=0.002).

**Conclusion:**

Patients with node-positive PHC might benefit from less aggressive approaches being associated with lower morbidity and a higher chance for adjuvant chemotherapy. Lymph node sampling might help to guide patients to the appropriate surgical approach according to their lymph node status.

**Supplementary Information:**

The online version contains supplementary material available at 10.1007/s00423-021-02154-4.

## Introduction

In perihilar cholangiocarcinoma (PHC), major hepatectomy is considered the gold standard beside experimental surgical therapies, such as liver transplantation (e.g., product-002 trial, DRKS00013276) [1, 2]. Tumor patterns with intraductal- and periductal-infiltrating growth often dictate the surgical strategy, favoring either right- or left-sided approaches. However, extended right hepatectomy should be preferred from an oncological point of view whenever technically feasible. This is due to the anatomical distance of the left hepatic artery to the tumor-bearing area and late segmental ramification of the left hepatic duct, both allowing wide resection margins [3]. Additionally, the left portal vein can easily be reconstructed, enabling a resection in *no-touch technique*, as firstly postulated by Peter Neuhaus [4, 5]. Besides others, these considerations account for the proposed survival benefit of right extended hepatectomy in PHC [5, 6].

While extended right hepatectomy guarantees excellent long-term survival, there is a substantial number of patients who do not necessarily benefit from radical surgery [7, 8]. In this context, it should be noted that the morbidity and mortality of this approach clearly exceed that of other hepatobiliary and pancreatic operations [5, 6]. Consequently, patients must be identified who do not benefit from extensive surgery in terms of long-term survival. In extended left hepatectomy, oncological compromises are often inevitable, e.g., the dissection of the right hepatic artery from the tumor. However, this approach is associated with lower morbidity and mortality and may, therefore, represent a valuable alternative, at least for some patients.

Lymphatic metastasis is quite commonly seen in patients with PHC and associated with significantly poorer prognosis after surgery [9–12]. Consequently, lymph node sampling is mandatory before liver transplantation, and lymph node metastases are considered a contraindication (e.g., product-002 trial, DRKS00013276). Regardless of listing policies before liver transplantation, the lymph node status does neither change the surgical strategy in liver resection [13, 14] nor does it affects the question of whether patients should be scheduled for adjuvant chemotherapy [15]. The aim of this study was to assess prognostic factors after liver resection in PHC and to investigate whether the advantages of established surgical concepts do also apply to node-positive patients.

## Methods

### Patients

All consecutive patients undergoing major hepatectomy for PHC between January 2005 and December 2015 at the Department of Surgery, Campus Charité – Mitte and Campus Virchow Klinikum, Charité – Universitätsmedizin Berlin were retrospectively analyzed. The primary patient outcome parameter was mean overall survival (mOS). This retrospective study was approved by the local ethics committee (EA2/006/16).

Besides survival rates, the database included demographic data such as gender, age, American Association of Anesthesiologists (ASA) score, and body mass index (BMI). Further, intra- and perioperative details, including postoperative morbidity (according to Dindo-Clavien [16]), Bismuth-Corlette classification (based on postoperative histopathology reports [17]), 30- and 90-day mortality, perioperative transfusion of blood products, as well as the length of stay (LOS) and length intensive care unit stay (ICU-LOS), were recorded.

### Preoperative management

Preoperative management was highly individualized and routinely included computed tomography and/or magnetic resonance imaging of the chest and abdomen, endoscopic retrograde cholangiography (ERC) with biliary stenting, or percutaneous transhepatic cholangiodrainage (PTCD). Determination of carbohydrate antigen 19-9 (CA 19-9) and carcinoembryonic antigen (CEA) ideally supplemented the work-up. In patients with suspicion of peritoneal carcinomatosis, either diagnostic laparoscopy or laparotomy was performed.

### Surgical procedure

The analysis included all cases of major hepatectomy. The distinction was made with regard to the side, e.g., right vs. left hepatectomy. Since segment 1 was resected in all cases as well as parts of segment 4, hepatectomies are classified as extended hemihepatectomies which were differentiated from left and right trisectionectomies. In hilar en bloc resections, lymphadenectomy is performed strictly on the left of the liver hilum, towards the upper pancreatic margin, and the coeliac artery. In selected cases, retropancreatic lymph nodes were dissected as well. The perihilar nodes are not dissected; these are retrieved en bloc with the resected specimen. Left hepatectomy and standard major hepatectomy procedures are performed accordingly with the difference that perihilar lymph nodes cannot be removed en bloc and have to be dissected and seperated from the bile duct bifurcation [5]. Approaches with technical modifications, such as segment-4 preserving variations [18], portal vein, and hepatic artery resection, were included but not further differentiated for statistical reasons. Extrahepatic bile duct resection alone on the one hand and multivisceral resections, e.g., hepatoduodenopancreatectomy (HPD), on the other hand, were excluded from the analysis. Patients with intrahepatic or distant metastases, as well as local peritoneal carcinomatosis, who underwent hepatectomy in individualized concepts, were excluded from the analysis as well.

### Histopathology

In all cases, PHC was confirmed according to the histopathological reports of the resected specimen. Our database included details from the histopathological reports, including TNM (8th edition, [19]) and UICC stage, R status, L status, grading, and microvascular infiltration.

### Follow-up

Patients were followed up in the outpatient clinic or with their general practitioner. Patients routinely underwent regular check-ups, including testing of CA 19-9 serum levels and abdominal ultrasound, CT, or MRI. We furthermore recorded whether adjuvant chemotherapy was performed. Adjuvant chemotherapy was not routinely recommended in patients until adjuvant chemotherapy had been shown to prolong overall survival (BILCAP trial [15]). Decisions to recommend adjuvant chemotherapy were made on an individual basis and generally included either gemcitabine (± cisplatin) or fluorouracil/capecitabine.

### Statistics

IBM SPSS Statistics for Macintosh Version 24.0 (IBM Corp., Armonk, NY, USA) was used for all calculations. R Studio Version 1.2.5033 (R Studio, Boston, MA, USA) was used for propensity score matching analysis. *p* values < 0.05 were considered significant.

Continuous parameters are reported as median and range. Counts and proportions are shown for categorical variables. Continuous variables were analyzed with the non-parametric Wilcoxon rank-sum test. The Pearson χ2 test was used for all categorical variables. Kaplan-Meier analysis of 1-year and 3-year and 5-year survival was performed and compared using the log-rank test. Results are reported in cumulative proportions at the end of each year. A conditional forward Cox regression model was created with all significant influencing factors of the univariate analysis. Results of the Cox regression are reported as hazard ratio (HR) and 95% confidence interval (95% CI). Factors were subsequently included in the multivariate cox regression model if the *p*-value was below 0.10 in univariate analysis.

Among lymph node positive patients, patients’ characteristics were compared according to the side of hepatic resection. Differentiating factors were integrated into a multivariate propensity score matching analysis — this included age, L status, and T stage. In logistic regression, a score was created, and patients matched through nearest neighbor matching with a caliper of 0.20.

## Results

### Patients’ characteristics

Two hundred and sixty patients underwent major hepatectomy for PHC between 2005 and 2015, of which 231met the inclusion criteria. Bilateral involvement of the second-order intrahepatic bile ducts, classified as Bismuth-Corlette type IV, was evident in 100 patients (43%). Table 1 shows all patient characteristics. One hundred and nine patients (47%) had histopathologically confirmed local lymph node metastases. Node-positive patients tended to show characteristics of more advanced tumors, including microvascular invasion, histopathological grading, perineural sheath infiltration, L status, T stage, and CA 19-9 levels.

**Table 1:** Patient characteristics

	Resected perihilar cholangiocarcinoma
	*n* = 231
Age ^1^	65 (33–83)
BMI ^1^	24.5 (16–41)
Gender (male) ^2^	156 (60)
ASA score ^2^	
1	12 (5)
2	130 (56)
3	85 (37)
4	4 (2)
Bismuth-Corlette ^2^	
I	8 (4)
II	17 (7)
IIIa	55 (24)
IIIb	44 (19)
IV	100 (43)
UICC stage ^2^	
I	10 (4)
II	79 (34)
IIIa	31 (13)
IIIb	104 (45)
IVa	7 (3)
IVb	0 (0)
Resection margin ^2^	
R0	154 (68)
R1	73 (32)
Lymph node status ^2^	
N0	122 (53)
N+	109 (47)
Microvascular invasion ^2^	
Yes	41 (20)
No	160 (80)
Histopathological grading ^2^	
Grade 1	11 (5)
Grade 2	153 (67)
Grade 3	63 (28)
Perineural sheath infiltration ^2^	
Yes	164 (90)
No	20 (11)
Lymphangitis carcinomatosa ^2^	
Yes	89 (46)
No	106 (54)
T Stage ^2^	
1	16 (7)
2a	63 (27)
2b	67 (29)
3	78 (34)
4	7 (3)
Resection side ^2^	
Left hepatectomy	86 (37)
Extended left hepatectomy	15 (7)
Left trisectionectomy	71 (31)
Right hepatectomy	145 (63)
Extended right hepatectomy	6 (3)
Right trisectionectomy	139 (60)
Surgical approach ^2^	
Standard major hepatectomy	111 (48)
Hilar en bloc resection	120 (52)
Portal vein resection ^2^	
Yes	136 (59)
No	95 (41)
Complications (Clavien-Dindo) ^2^	
None	28 (12)
I	11 (5)
II	51 (22)
IIIa	62 (27)
IIIb	41 (18)
IVa	6 (3)
IVb	1 (0)
V	31 (12)
CA 19-9 (kU/l) ^1^	63.0 (1–32670)
ICU stay (days) ^1^	4 (2–123)
Hospital stay (days) ^1^	23.0 (7–213)
30-day mortality ^2^	16 (7%)
90-day mortality ^2^	29 (13)
Hospital readmission	48 (21)
Adjuvant chemotherapy	
Yes	39 (18)
No	182 (82)
Recurrence / Death ^2^	176 (76)

1 Data is presented as median and range. 2 Data is presented as count and proportions (%)

### Approaches and postoperative morbidity and mortality

In two-thirds of all patients (63%), an extended right hepatectomy was performed, of which 77% were performed as a formal *hilar en bloc* resection (Table 1). Accordingly, 37% underwent extended left hepatectomy. When analyzed according to the Bismuth classification, a right-sided resection was performed in 75% of all Bismuth I (*n*=6), 71% of all Bismuth II, in 89% of all Bismuth IIIa, in 27% of all Bismuth IIIb (*n*=12), and in 61% of all Bismuth IV tumors (*n*=61). Major postoperative complications, as defined by Dindo-Clavien IIIa–V, occurred in 61% of all patients, with significantly more complications being noted after extended right hepatectomy (67%) when compared to extended left hepatectomy (33%, *p* = 0.048). Thirty-day and 90-day mortality was 7% and 13%, respectively. Both 30-day (11% vs. 0%, *p* = 0.001) and 90-day mortality (18% vs. 4%, *p*=0.001) were significantly higher after extended right hepatectomy, when compared to extended left hepatectomy. Of note, major complications (70% vs. 53%, *p* = 0.011) were more frequently seen, and 90-day mortality (20% vs. 6%) was significantly higher in lymph node-positive patients when compared to lymph node-negative patients (all *p* < 0.05, Supplementary Table S1). In node-positive patients, there was also a trend towards more major complications (75% vs. 61%, *p* = 0.126) and a significantly higher 30-day (16% vs. 0%, *p* = 0.010) and 90-day mortality (27% vs. 8%, *p* = 0.019), respectively, after extended right hepatectomy. Table 1 provides an overview of all clinical data.

## Tumor-free margins after extended right and left hepatectomy

Considering 90-day mortality after extended right hepatectomy of ~ 30% in lymph node-positive patients, we aimed to investigate whether postulated oncological benefits of extended right hepatectomy do also apply to the subgroup of lymph node positive patients.

Tumor-free resection margins could be achieved in 68% of all patients, with a trend towards a higher rate of R0 after extended right hepatectomy when compared to extended left hepatectomy (R0, 72% vs. 62%, *p* = 0.147; Supplementary Table S2). The superiority of extended right hepatectomy over extended left hepatectomy with regard to microscopically free margins reached statistical significance in lymph node-negative patients (N0, extended right hepatectomy: 86% vs. extended left hepatectomy: 66%, *p* = 0.008). In lymph node-positive patients, there were no differences noted between extended right and left hepatectomy with regard to microscopically tumor-free margins (N1, extended right hepatectomy: 55% vs. extended left hepatectomy: 58%, *p* = 0.778; Supplementary Table S2). Comparing patients according to their lymph node status, microscopically tumor-free margins were generally less common in lymph node-positive patients (R0, 22% vs. 44%, *p* < 0.001). Microscopically tumor-free margins were more likely achieved, if extended hepatectomy was combined with *hilar en bloc* resection (90% vs. 67%, *p* = 0.003) and portal vein resection (86% vs. 69 %, *p* = 0.018), respectively. Again, this was not true for the subgroup of node-positive patients. Locally radical approaches seem less promising in patients with positive lymph nodes (Supplementary Table S2).

### Survival in lymph node positive patients according to resection margin

The superiority of extended right hepatectomy with regard to microscopically negative margins is obviously limited to lymph node negative patients. We were next interested in the question of whether microscopically negative margins were indeed associated with improved survival in lymph node positive patients.

**Fig. 1:**
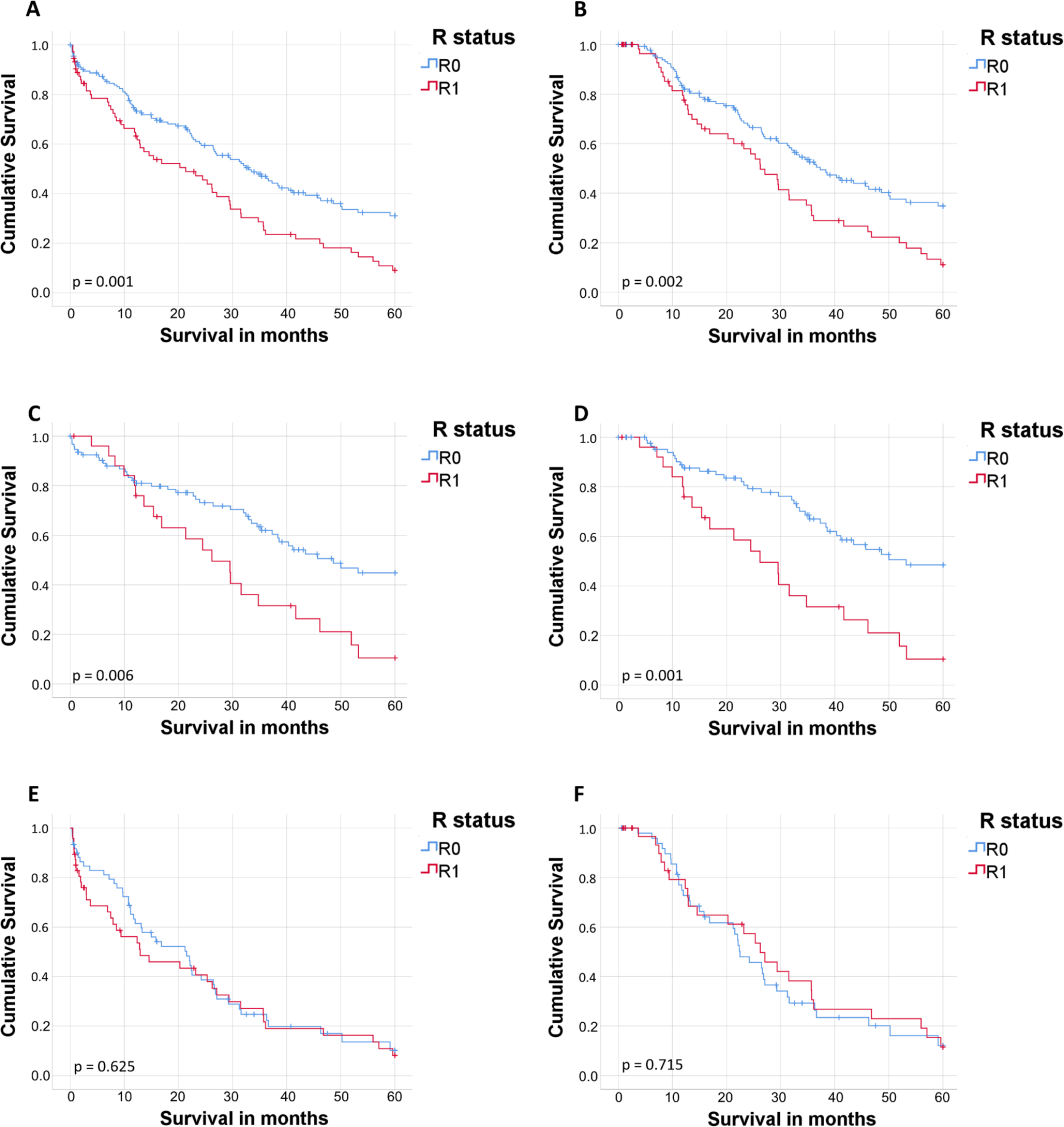
Overall survival according to lymph node and R status. Kaplan Meier curves of overall survival. **A** All resected patients with perihilar cholangiocarcinoma with and without lymph node metastases (N0 and N+) according to R status. **B** All resected patients with perihilar cholangiocarcinoma with and without lymph node metastases (N0 and N+) excluding 90-day mortality according to R status. **C** Resected patients with perihilar cholangiocarcinoma without lymph node metastases (N0) according to R status. **D** Resected patients with perihilar cholangiocarcinoma without lymph node metastases (N0) excluding 90-day mortality according to R status. **E** Resected patients with perihilar cholangiocarcinoma with lymph node metastases (N+) according to R status

In all patients, irrespective of the lymph node status and in lymph node-negative patients, mOS (Fig. 1a) was significantly higher when microscopically tumor-free margins could be achieved (N0/N+: 49.4 vs. 27.2 months, *p* = 0.001, Fig. 1a; N0: 63.8 vs. 33.7 months, *p* = 0.006, Fig. 1c). However, there were no such differences between R0 and R1 noted in lymph node-positive patients (mOS, 25.0 vs. 23.2 months, *p* = 0.625, Fig. 1e). Five-year survival rates, excluding 90-day mortality, are shown in Fig. 1 b, d, and f.

**Fig. 2:**
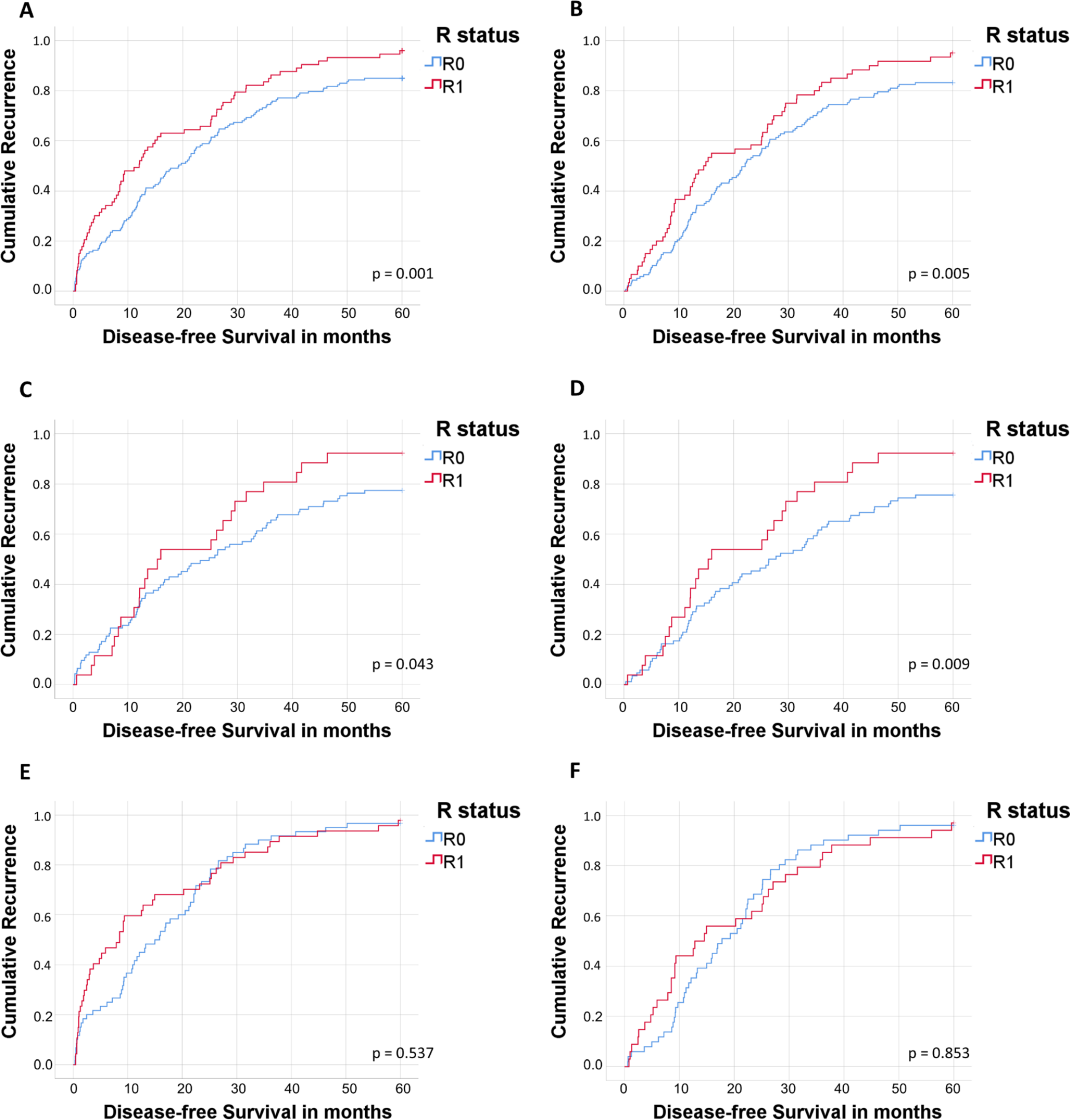
Disease-free survival according to lymph node and R status. Kaplan Meier curves of disease-free survival. **A** All resected patients with perihilar cholangiocarcinoma with and without lymph node metastases (N0 and N+) according to R status. **B** All resected patients with perihilar cholangiocarcinoma with and without lymph node metastases (N0 and N+) excluding 90-day mortality according to R status. **C** Resected patients with perihilar cholangiocarcinoma without lymph node metastases (N0) according to R status. **D** Resected patients with perihilar cholangiocarcinoma without lymph node metastases (N0) excluding 90-day mortality according to R status. **E** Resected patients with perihilar cholangiocarcinoma with lymph node metastases (N+) according to R status. **F** Resected patients with perihilar cholangiocarcinoma with lymph node metastases (N+) excluding 90-day mortality according to R status

In accordance, DFS was significantly higher in patients, irrespective of the lymph node status, and in lymph node-negative patients, when microscopically tumor-free margins could be achieved (N0/N+: 40.8 vs. 22.0 months, *p* = 0.002, Fig. 2a; N0: 53.5 vs. 30.3 months, *p* = 0.043, Fig. 2c). Again, among lymph node-positive patients, DFS did not differ significantly between R0 and R1 (19.8 vs. 17.6 months, *p* = 0.537, respectively). Five-year disease-free survival rates, excluding 90-day mortality, are shown in Fig. 2 B, D, and F. The cumulative 1-, 3-, and 5-year survival rates and disease-free survival rates according to N and R status are displayed in Supplementary Table S3.

Long-term survival in lymph node-positive patients after extended right and left hepatectomy

Node-positive patients obviously do not benefit from achieving microscopically tumor-free margins. We, therefore, investigated next whether this subgroup of patients might benefit from less aggressive approaches being associated with lower morbidity.

Mean overall survival (mOS) for all patients was 41.4 months (N0, 56.4 vs. N+, 24.4, *p* < 0.001); the cumulative 1-, 3-, and 5-year survival rates were 72% (N0: 82%, N+: 62%), 48% (N0: 62%, N+: 32%), and 36% (N0: 49%, N+: 22%), respectively. Mean disease-free survival (DFS) was 34.4 months (N0-patients: 48.3 months vs. N± patients: 18.9 months, *p* < 0.001); the cumulative 1-, 3-, and 5-year DFS rates were 60% (N0: 69%, N+ 50%), 22% (N0: 32%, N+: 11%), and 12% (N0: 20%, N+: 3%), respectively.

**Fig. 3:**
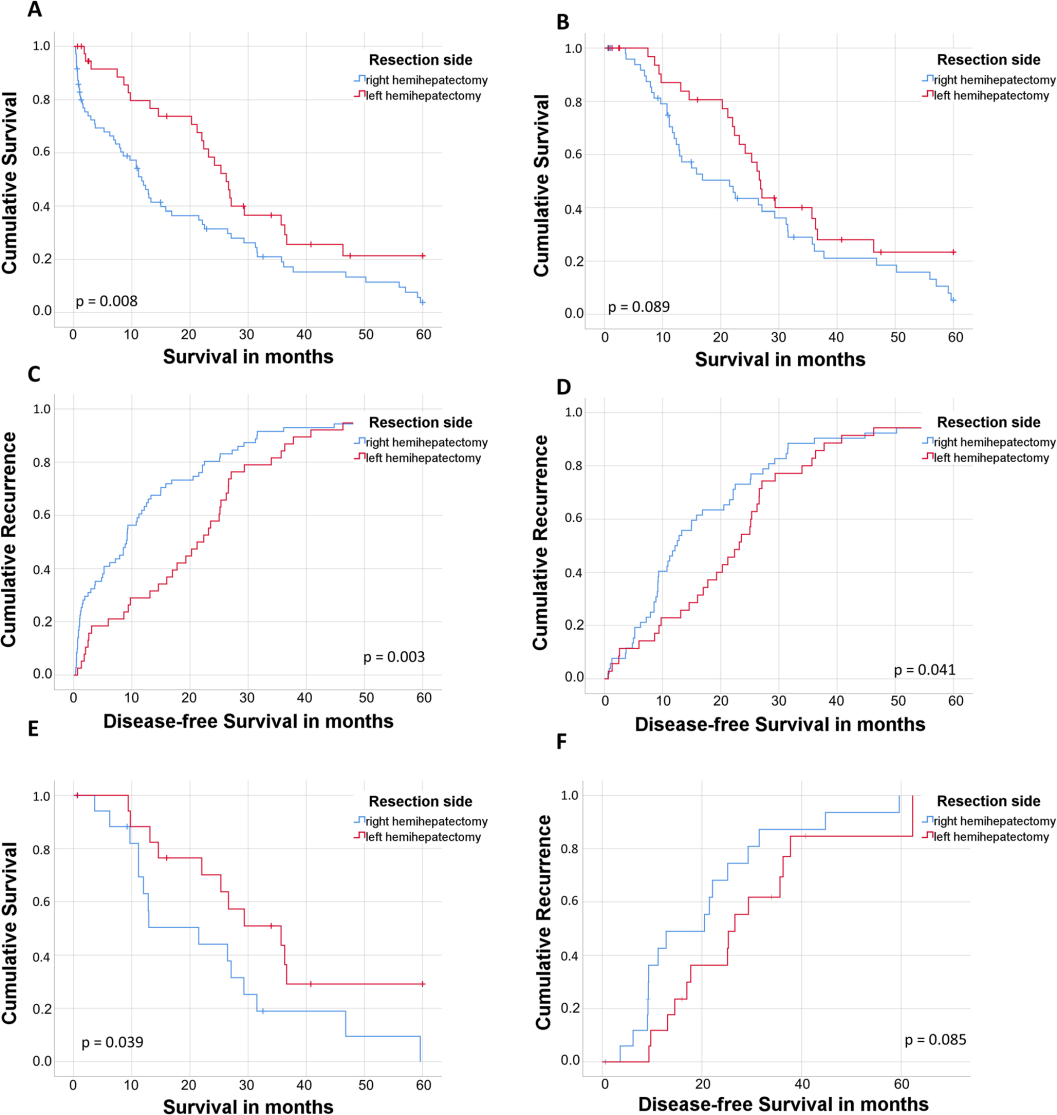
Overall and disease-free survival of patients with lymph node positive perihilar cholangiocarcinoma according to side of hepatic resection. Kaplan Meier curves of patients with lymph node metastases and resected perhilar cholangiocarcinoma according to side of hepatic resection. A. Overall survival. B. Overall survival excluding 90-day mortality. C. Disease-free survival. D. Disease-free survival excluding 90-day mortality. E. Overall survival after propensity score matching excluding 90-day mortality. F. Disease-free survival after propensity score matching excluding 90-day mortality.

In the subgroup of lymph node-positive patients, cumulative 1-, 3-, and 5-year survival after extended right and left hepatectomy were 52% and 82% (*p* = 0.002), 27% and 42% (*p* = 0.102), and 16% and 34% (*p* = 0.025), respectively (Fig. 3a). Figure 3 b shows the survival curve excluding 90-day mortality. The cumulative 1-, 3-, and 5-year DFS in lymph node-positive patients for right and left extended hepatectomy were 38% and 71% (*p* = 0.001), 9% and 16% (*p* = 0.243), 1% and 5% (*p* = 0.241), respectively (Fig. 3c). Figure 3 d shows the DFS excluding 90-day mortality.

Extended left hepatectomy had lower morbidity (Supplementary Table S4) as well as the superior long-term outcome when compared to extended right-sided resections in lymph node-positive patients. After excluding 90-day mortality, the benefit was still evident but short of statistical significance. After propensity score matching for T stage and L status, the difference was statistically significant (Fig. 3 e and f).

### Prognostic factors determining long-term survival

Univariate Cox regression analysis revealed that T stage < 3, histopathological grading, N status, R status, L status, and V status were variables of prognostic significance. In multivariate analysis, R, N, and V status were found to be independent prognostic factors (Supplementary Table S5). When only considering lymph node-negative patients, the univariate cox regression analysis showed that T stage < 3, R status, and V status were of prognostic value. In multivariate analysis, R status was the only independent variable with prognostic significance in those patients (Supplementary Table S5). In lymph node-positive patients, histopathological grading, V status, and adjuvant chemotherapy were associated with OS in univariate analysis. Multivariate Cox Regression showed that only grading and adjuvant chemotherapy were independently associated with OS (Supplementary Table S5). In contrast to lymph node-negative patients, R status was not independently associated with OS in lymph node positive patients.

## Discussion

Major hepatectomy aiming for microscopically tumor-free margins represents the only potentially curative therapy in PHC. Surgical strategies are often determined by patterns of tumor growth, such as vascular infiltration or atrophy of one liver lobe. Whenever technically possible, extended right hepatectomy should be preferred due to its oncologic superiority. From our personal perspective, this should ideally be performed in *hilar en bloc* technique [4, 5]. Extended left hepatectomy is associated with technical compromises, at least in parts due to the anatomical relationship of the right hepatic artery and the tumor-bearing area. Indeed, we could confirm many findings from previous studies for the whole cohort of patients [4, 12, 20]. Our cohort was characterized by a high percentage of locally advanced tumors. Bismuth IV tumors were most frequently seen, and positive lymph nodes were evident in almost half of the patients. Among node-negative patients, extended right hepatectomy was associated with a significantly higher chance for microscopically tumor-free margins. The superiority of extended right hepatectomy could be further increased by applying the *hilar en bloc* technique. This is of importance as microscopically tumor-free resection margins were confirmed to be the only independent prognostic factor that can be influenced by the surgical strategy. However, convincing results were mainly due to effects in the group of lymph node-negative patients. Patients without lymph node metastases may indeed benefit most from the locally aggressive surgical approaches, at least from a conceptual oncological point of view.

Overall postoperative (90-day) mortality was 13% in the present study. This is in line with the mortality rates of a recently published multicenter study with 12% (no portal vein embolization) and 18% (after portal vein embolization) mortality [21]. On the other hand, Nagino and colleagues report mortality rates ranging between 2 and 5% ([22–24]), which is lower when compared to the numbers in the present study. However, due to significant differences in patient characteristics such as age and comorbidities, the comparison of mortality rates between eastern and western centers is difficult [25]. Nevertheless, the 90-day mortality of ~ 30% after extended right hepatectomy in the subset of lymph node-positive patients must be well-founded in view of accepted mortality rates in HPB surgery. Given an 8% mortality after extended left hepatectomy, less aggressive approaches appear particularly appealing in lymph node-positive patients. Another finding supporting this hypothesis is the fact that more patients with advanced UICC stages underwent left hepatectomy while achieving better long-term survival than patients who underwent right-sided resections.

In this respect, it should be considered that node-positive patients do obviously not benefit from microscopically tumor-free margins. Multivariate analysis revealed that adjuvant chemotherapy was, besides V status, the only independent prognostic factor determining long-term survival. These findings are supported by the BILCAP study, suggesting lymph node positive patients benefit more from adjuvant chemotherapy [15]. These findings indicate that established surgical concepts are of minor importance in node-positive patients with regard to long-term survival. Instead, these patients benefit most from surgical approaches with low postoperative morbidity, as this increases the chance for adjuvant chemotherapy [26].

**Fig. 4:**
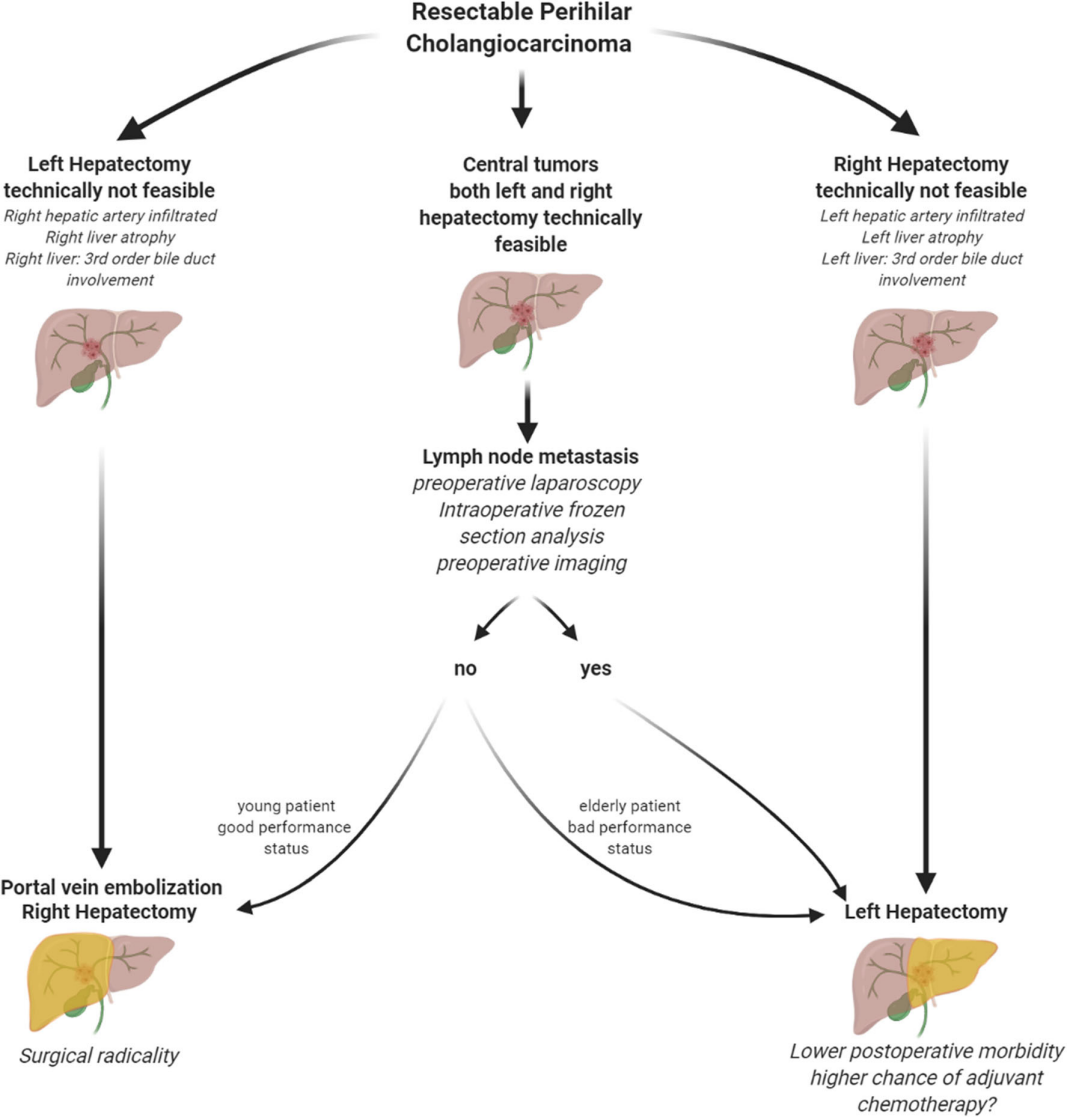
Suggested therapy algorithm in resectable perihilar cholangiocarcinoma Suggested tailored approach in patients diagnosed with resectable perihilar cholangiocarcinoma without distant metastases (Figure created with Biorender.com)

Therefore, we suggest a new pathway according to the patients’ lymph node status, which is shown in Fig. 4. Based on the results found in the present study, we propose that for patients with central PHC (both left and right hepatectomy technically feasible, central column in Fig. 4), a thorough evaluation of the patients’ lymph node status should be performed before resection. This should primarily be achieved based on preoperative imaging. An analysis conducted by Ruys et al. [80] found a sensitivity of 61% and specificity of 88% in detecting nodal metastases [27]. Similar to PHC patients who are scheduled for liver transplantation (e.g., product-002 trial, DRKS00013276), the assessment of the patients’ nodal status can be completed by a preoperative open or laparoscopic sampling of lymph nodes around the coeliac/common hepatic artery, upper pancreatic margin, as well as retroduodenal nodes. Especially macroscopically suspicious lymph nodes should be examined. However, the perihilar region should not be dissected for oncological reasons. In case lymph node metastases are detected and/or the patient has a bad performance status, a left hepatectomy procedure should be performed. In young and fit patients without the presence of lymph node metastases, we recommend right trisectionectomy with hilar en bloc resection as reported by Neuhaus et al. [5].

There are obvious limitations to the present study, including its retrospective design as well as the use of data from a single-center leading to potential bias. Furthermore, preoperative assessment of the patients’ lymph node status, either by preoperative imaging or staging laparoscopy/laparotomy, may be inaccurate or inconclusive in some patients which.

Future prospective analyses focusing on the surgical approach with regards to the patients’ lymph node status should be performed to strengthen the results found in the present study and support the suggested pathway according to the patients’ lymph node status (Fig. 4).

## Conclusions

Node-positive patients might benefit from less extensive surgery, which is associated with lower morbidity and the opportunity for improved survival benefits of multimodal therapy. Taking into account the lymph node status before determining the resection side might help to identify patients who really benefit from radical surgical approaches, such as extended right hepatectomy.

## Supplementary Information

ESM 1 (DOCX 41 kb)

## Data Availability

All data are available from the corresponding author on reasonable request.
