# Correction to: A tailored approach in lymph node-positive perihilar cholangiocarcinoma

**DOI:** 10.1007/s00423-021-02332-4

**Published:** 2021-10-31

**Authors:** Christian Benzing, Felix Krenzien, Alexa Mieg, Annika Wolfsberger, Andreas Andreou, Nora Nevermann, Uwe Pelzer, Uli Fehrenbach, Lena Marie Haiden, Robert Öllinger, Wenzel Schöning, Moritz Schmelzle, Johann Pratschke

**Affiliations:** 1 Department of Surgery, Campus Charité Mitte Campus Virchow-Klinikum, Experimental Surgery and Regenerative Medicine, Charité – Universitätsmedizin Berlin, Berlin, Germany; 2 Department of Hematology, Oncology and Tumor Immunology, Charité – Universitätsmedizin Berlin, Berlin, Germany; 3 Department of Radiology, Charité - Universitätsmedizin Berlin, Berlin, Germany

**Correction to** **:** **Langenbeck's Archives of Surgery (2021) 406:1499–1509**

**https://doi.org/10.1007/s00423-021-02154-4**

Figures 3 and 4 did not precisely match the text and were incorrect. Figure 3 refers to the survival according to the side of hepatic resection and not R-status. In Figure 4, the reasons for left/right hepatectomy not being feasible were reversed.

Corrected figures and figure legends are shown below:

Figure 3: Overall and disease-free survival of patients with lymph node positive perihilar cholangiocarcinoma according to side of hepatic resection

Kaplan Meier curves of patients with lymph node metastases and resected perhilar cholangiocarcinoma according to side of hepatic resection. A. Overall survival. B. Overall survival excluding 90-day mortality. C. Disease-free survival. D. Disease-free survival excluding 90-day mortality. E. Overall survival after propensity score matching excluding 90-day mortality. F. Disease-free survival after propensity score matching excluding 90-day mortality.
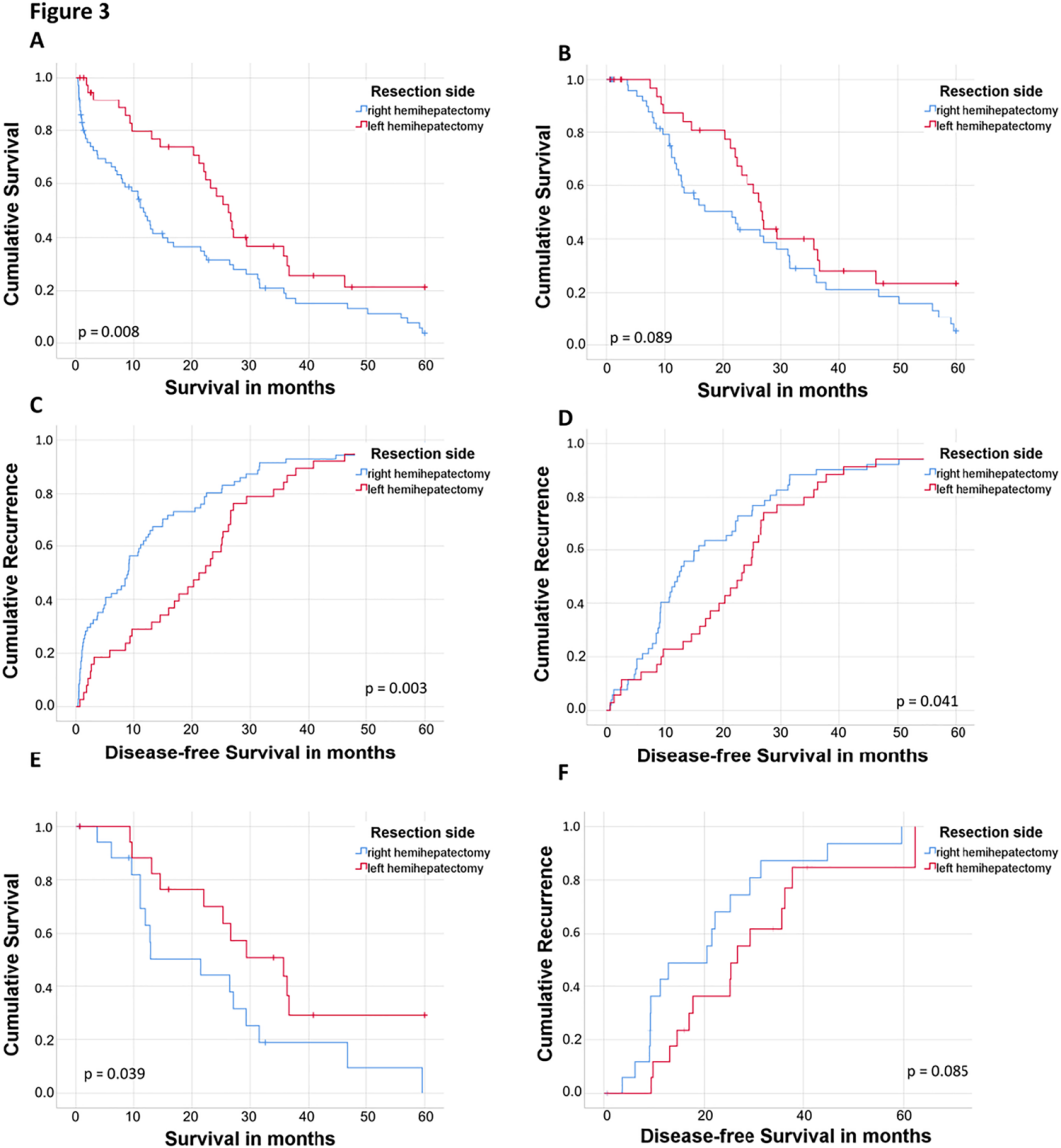


Figure 4: Suggested therapy algorithm in resectable perihilar cholangiocarcinoma

Suggested tailored approach in patients diagnosed with resectable perihilar cholangiocarcinoma without distant metastases (Figure created with Biorender.com)
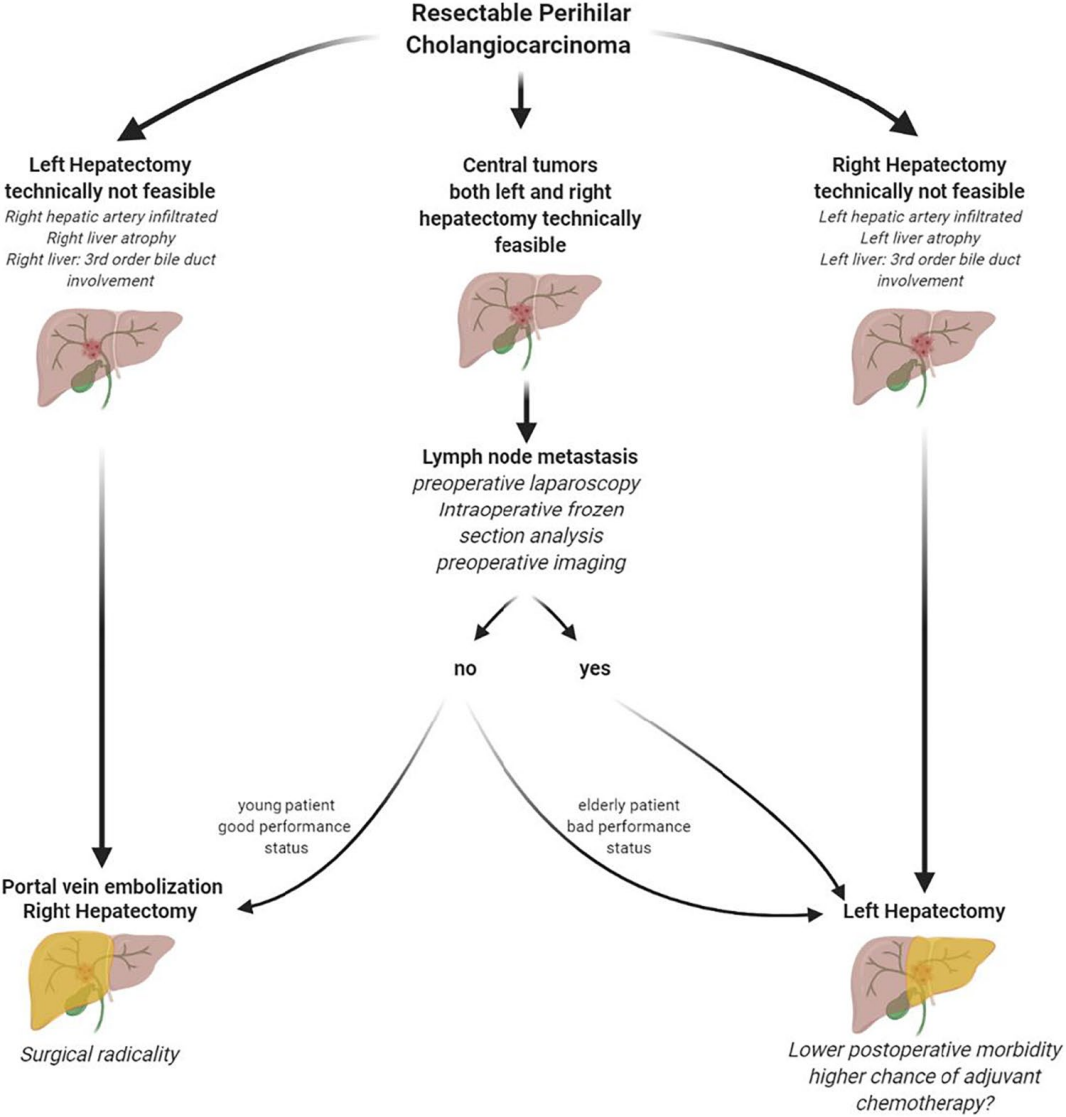


The original article has been corrected

