# Supplementary material for: A tailored approach in lymph node-positive perihilar cholangiocarcinoma
Source: Langenbecks Arch Surg. 2021 Jun 1;406(5):1499–509. doi: 10.1007/s00423-021-02154-4 (PMC8370897; doi:10.1007/s00423-021-02154-4)
Supplement: Supplementary file 1 — (DOCX 41 kb) [file 423_2021_2154_MOESM1_ESM.docx]

**Supplementary Table S1.** Patient characteristics according to lymph node status

|  | N0 | N+ | *P* value |
| --- | --- | --- | --- |
|  | n = 122 | n = 109 |  |
| Age ^1^ | 66 (34-83) | 64 (33-83) | 0.424 |
| BMI ^1^ | 24.2 (18-38) | 24.8 (16-41) | 0.491 |
| Gender (male) ^2^ | 72 (59) | 67 (62) | 0.704 |
| ASA score ^2^ |  |  | 0.201 |
| 1 | 6 (5) | 6 (6) |  |
| 2 | 70 (57) | 60 (55) |  |
| 3 | 46 (38) | 39 (36) |  |
| 4 | 0 (0) | 4 (4) |  |
| Bismuth-Corlette ^2^ |  |  | 0.967 |
| I | 5 (4) | 3 (3) |  |
| II | 8 (7) | 9 (8) |  |
| IIIa | 29 (25) | 26 (24) |  |
| IIIb | 23 (20) | 21 (20) |  |
| IV | 51 (44) | 49 (45) |  |
| UICC Stage ^2^ |  |  | <0.001 |
| I | 10 (8) | 0 (0) |  |
| II | 79 (65) | 0 (0) |  |
| IIIa | 31 (25) | 0 (0) |  |
| IIIb | 0 (0) | 104 (95) |  |
| IVa | 2 (2) | 5 (5) |  |
| Resection margin ^2^ |  |  | <0.001 |
| R0 | 94 (78) | 60 (56) |  |
| R1 | 26 (22) | 47 (44) |  |
| Microvascular invasion ^2^ |  |  | 0.020 |
| Yes | 15 (14) | 26 (63) |  |
| No | 91 (86) | 69 (73) |  |
| Histopathological grading ^2^ |  |  | 0.030 |
| Grade 1 | 7 (6) | 4 (4) |  |
| Grade 2 | 90 (74) | 63 (60) |  |
| Grade 3 | 25 (21) | 38 (36) |  |
| Perineural sheath infiltration ^2^ |  |  | 0.046 |
| Yes | 76 (84) | 88 (94) |  |
| No | 14 (16) | 6 (6) |  |
| Lymphangitis carcinomatosa ^2^ |  |  | <0.001 |
| Yes | 74 (73) | 62 (66) |  |
| No | 27 (27) | 32 (34) |  |
| T Stage ^2^ |  |  | 0.009 |
| 1 | 10 (8) | 6 (6) |  |
| 2a | 43 (35) | 20 (18) |  |
| 2b | 36 (30) | 31 (28) |  |
| 3 | 31 (25) | 47 (43) |  |
| 4 | 2 (2) | 5 (5) |  |
| Resection side ^2^ |  |  | 0.515 |
| Left hepatectomy | 48 (39) | 38 (35) |  |
| Extended left hepatectomy | 7 (6) | 8 (7) |  |
| Left trisectionectomy | 41 (34) | 30 (38) |  |
| Right hepatectomy | 74 (61) | 70 (65) |  |
| Extended right hepatectomy | 3 (3) | 3 (3) |  |
| Right trisectionectomy | 71 (58) | 68 (62) |  |
| Surgical approach ^2^ |  |  | 0.248 |
| Standard major hepatectomy | 63 (52) | 48 (43) |  |
| Hilar en bloc resection | 59 (48) | 61 (51) |  |
| Portal vein resection ^2^ |  |  | 0.119 |
| Yes | 66 (54) | 70 (64) |  |
| No | 56 (46) | 39 (36) |  |
| Complications (Clavien-Dindo) ^2^ |  |  | 0.018 |
| None | 18 (15) | 10 (9) |  |
| I | 10 (8) | 1 (1) |  |
| II | 29 (23) | 22 (20) |  |
| IIIa | 33 (27) | 29 (27) |  |
| IIIb | 19 (16) | 22 (20) |  |
| IVa | 3 (3) | 3 (3) |  |
| IVb | 1 (1) | 0 (0) |  |
| V | 9 (7) | 22 (20) |  |
| CA 19-9 (kU/l) ^1^ | 34.4 (1-32670) | 176 (1-23049) | 0.002 |
| ICU stay (days) ^1^ | 3 (2-123) | 5 (2-111) | 0.016 |
| Hospital stay (days) ^1^ | 22 (7-185) | 26 (9-213) | 0.241 |
| 90-day mortality ^2^ | 7 (6) | 22 (20) | 0.001 |
| Hospital readmission | 27 (22) | 21 (19) | 0.592 |
|  |  |  |  |
|  |  |  |  |
|  |  |  |  |
| Adjuvant Chemotherapy |  |  | 0.002 |
| Yes | 12 (10) | 76 (74) |  |
| No | 106 (90) | 27 (26) |  |
| Recurrence / Death ^2^ | 82 (67) | 94 (86) | 0.001 |

^1 Data is presented as median and range, 2 Data is presented as count and proportions (%)^

**Supplementary Table S2.** Resection margin status according to surgical approach and lymph node status

| All patients (N0/N+) patients | | | |
| --- | --- | --- | --- |
|  | R0 | R1 | *P* value |
|  | n = 154 | n = 72 |  |
| Resection side |  |  | 0.147 |
| Left Hepatectomy | 53 (62) | 32 (38) |  |
| Right Hepatectomy | 101 (72) | 40 (28) |  |
| Surgical approach |  |  | 0.134 |
| Standard major hepatectomy | 68 (63) | 40 (37) |  |
| Hilar en bloc resection | 86 (72) | 33 (28) |  |
| Portal vein resection |  |  | 0.485 |
| Yes | 94 (70) | 41 (30) |  |
| No | 60 (65) | 32 (35) |  |
|  |  |  |  |
| N0 patients | | | |
|  | R0 | R1 | *P* value |
|  | n = 97 | n = 30 |  |
| Resection side |  |  | 0.008 |
| Left Hepatectomy | 31 (66) | 16 (34) |  |
| Right Hepatectomy | 63 (86) | 10 (14) |  |
| Surgical approach |  |  | 0.003 |
| Standard major hepatectomy | 41 (67) | 20 (33) |  |
| Hilar en bloc resection | 53 (90) | 6 (10) |  |
| Portal vein resection |  |  | 0.018 |
| Yes | 57 (86) | 9 (14) |  |
| No | 37 (69) | 17 (31) |  |
|  |  |  |  |
| N+ patients | | | |
|  | R0 | R1 | *P* value |
|  | n = 60 | n = 47 |  |
| Resection side |  |  | 0.778 |
| Left Hepatectomy | 22 (58) | 16 (42) |  |
| Right Hepatectomy | 38 (55) | 31 (45) |  |
| Surgical approach |  |  | 0.800 |
| Standard major hepatectomy | 27 (57) | 20 (43) |  |
| Hilar en bloc resection | 33 (55) | 27 (45) |  |
| Portal vein resection |  |  | 0.491 |
| Yes | 37 (54) | 32 (46) |  |
| No | 23 (61) | 15 (40) |  |

**Supplementary Table S3.** Cumulative 1-, 3-, and 5-year survival rates according to resection margin status

|  | All patients (N0 and N+) | N0 | N+ |
| --- | --- | --- | --- |
|  | N = 231 | n = 122 | n = 109 |
| R0-Status |  |  |  |
| 1-year survival rate | 75% | 82% | 65% |
| 3-year survival rate | 53% | 67% | 32% |
| 5-year survival rate | 44% | 56% | 23% |
| 1-year disease-free survival rate | 64% | 69% | 57% |
| 3-year disease-free survival rate | 26% | 36% | 10% |
| 5-year disease-free survival rate | 16% | 23% | 3% |
| R1-Status |  |  |  |
| 1-year survival rate | 67% | 81% | 60% |
| 3-year survival rate | 34% | 39% | 32% |
| 5-year survival rate | 22% | 23% | 21% |
| 1-year disease-free survival rate | 51% | 69% | 40% |
| 3-year disease-free survival rate | 15% | 19% | 13% |
| 5-year disease-free survival rate | 4% | 8% | 2% |

**Supplementary Table S4.** Patient characteristics according to resection side

|  | All N+-patients | Right hemihepatectomy | Left hemihepatectomy | *P* value |
| --- | --- | --- | --- | --- |
|  | n=109 | n = 71 | n = 38 |  |
| Age ^1^ | 64 (33-83) | 64 (38-83) | 65.5 (33-83) | 0.552 |
| BMI ^1^ | 24.8 (16-41) | 24.0 (16-41) | 25.6 (19-37) | 0.098 |
| Gender (male) ^2^ | 67 (62) | 45 (63) | 22 (58) | 0.575 |
| ASA score ^2^ |  |  |  | 0.532 |
| 1 | 6 (6) | 5 (7) | 1 (3) |  |
| 2 | 60 (55) | 41 (58) | 19 (50) |  |
| 3 | 39 (36) | 23 (32) | 16 (42) |  |
| 4 | 4 (4) | 2 (3) | 2 (5) |  |
| Bismuth-Corlette ^2^ |  |  |  | <0.001 |
| I | 3 (3) | 2 (3) | 1 (3) |  |
| II | 9 (8) | 7 (10) | 2 (5) |  |
| IIIa | 26 (24) | 24 (34) | 2 (5) |  |
| IIIb | 21 (19) | 6 (9) | 15 (40) |  |
| IV | 49 (45) | 31 (44) | 18 (47) |  |
| UICC Stage ^2^ |  |  |  | 0.030 |
| I | 0 (0) | 0 (0) | 0 (0) |  |
| II | 0 (0) | 0 (0) | 0 (0) |  |
| IIIa | 0 (0) | 0 (0) | 0 (0) |  |
| IIIb | 104 (95) | 70 (99) | 34 (90) |  |
| IVa | 5 (5) | 1 (1) | 4 (11) |  |
| IVb | 0 (0) | 0 (0) | 0 (0) |  |
| Resection margin ^2^ |  |  |  | 0.778 |
| R0 | 60 (56) | 38 (55) | 22 (58) |  |
| R1 | 47 (44) | 31 (45) | 16 (42) |  |
| Microvascular invasion ^2^ |  |  |  | 0.883 |
| Yes | 26 (63) | 17 (28) | 9 (27) |  |
| No | 69 (73) | 44 (72) | 25 (74) |  |
| Histopathological grading ^2^ |  |  |  | 0.121 |
| Grade 1 | 4 (4) | 1 (2) | 3 (8) |  |
| Grade 2 | 63 (60) | 38 (57) | 25 (66) |  |
| Grade 3 | 38 (36) | 28 (42) | 10 (26) |  |
| Perineural sheath infiltration ^2^ |  |  |  | 0.083 |
| Yes | 88 (94) | 58 (91) | 30 (100) |  |
| No | 6 (6) | 6 (9) | 0 (0) |  |
| Lymphangitis carcinomatosa ^2^ |  |  |  | 0.111 |
| Yes | 62 (66) | 45 (73) | 18 (56) |  |
| No | 32 (34) | 17 (27) | 14 (47) |  |
| T Stage ^2^ |  |  |  | 0.009 |
| 1 | 6 (6) | 3 (4) | 3 (8) |  |
| 2a | 20 (18) | 15 (21) | 5 (13) |  |
| 2b | 31 (28) | 15 (21) | 16 (42) |  |
| 3 | 47 (43) | 37 (52) | 10 (26) |  |
| 4 | 5 (5) | 1 (1) | 4 (11) |  |
| T Stage ^2^ |  |  |  | 0.541 |
| <3 | 26 (24) | 18 (26) | 8 (21) |  |
| ≥3 | 83 (76) | 52 (74) | 31 (80) |  |
| Surgical approach ^2^ |  |  |  |  |
| Standard major hepatectomy | 48 (44) | 10 (14) | 38 (100) | <0.001 |
| Hilar en bloc resection | 61 (57) | 61 (86) | 0 (0) |  |
| Portal vein resection ^2^ |  |  |  | <0.001 |
| Yes | 70 (64) | 63 (89) | 7 (18) |  |
| No | 39 (36) | 8 (11) | 31 (82) |  |
| Complications (Clavien-Dindo) ^2^ |  |  |  |  |
| None | 10 (9) | 5 (7) | 5 (13) | 0.118 |
| I | 1 (1) | 1 (1) | 0 (0) |  |
| II | 22 (20) | 12 (17) | 10 (26) |  |
| IIIa | 29 (27) | 19 (27) | 10 (26) |  |
| IIIb | 22 (20) | 12 (17) | 10 (26) |  |
| IVa | 3 (3) | 2 (3) | 1 (3) |  |
| IVb | 0 (0) | 0 (0) | 0 (0) |  |
| V | 22 (20) | 20 (28) | 2 (5) |  |
| CA 19-9 (kU/l) ^1^ | 176 (1-23049) | 204 (1-10633) | 81 (1-23049) | 0.229 |
| ICU stay (days) ^1^ | 5 (2-111) | 6 (2-111) | 2.5 (2-32) | 0.001 |
| Hospital stay (days) ^1^ | 26 (9-213) | 28 (10-148) | 21.5 (9-213) | 0.355 |
| 90-day mortality ^2^ | 22 (20) | 19 (27) | 3 (8) | 0.019 |
| Hospital readmission | 21 (19) | 10 (14) | 11 (28) | 0.077 |
|  |  |  |  |  |
|  |  |  |  |  |
|  |  |  |  |  |
| Adjuvant Chemotherapy |  |  |  | 0.022 |
| Yes | 27 (27) | 13 (19) | 14 (40) |  |
| No | 76 (74) | 55 (81) | 21 (60) |  |
| Recurrence / Death ^2^ | 94 (86) | 65 (92) | 29 (76) | 0.028 |

^1 Data is presented as median and range, 2 Data is presented as count and proportions (%)^

**Supplementary Table S5**. Univariate and multivariate analysis of factors influencing overall survival in all resected patients (N0 and N+), patients with N0- and N+ status, respectively.

| **All patients (N0 and N+)** | | | | | | |
| --- | --- | --- | --- | --- | --- | --- |
|  | Univariate | | | | Multivariate | |
| Variable | HR (95% CI) | | *P* value | | HR (95% CI) | *P* value |
| Age | 1.013 (0.997-1.029) | | 0.118 | |  |  |
| Gender (male) | 1.265 (0.916-1.747) | | 0.153 | |  |  |
| Body Mass Index | 1.022 (0.980-1.066) | | 0.303 | |  |  |
| Bismuth-Corlette > II | 0.836 (0.504-1.386) | | 0.488 | |  |  |
| T Stage <3 | 0.629 (0.460-0.862) | | 0.004 | | 0.738 (0.485-1.123) | 0.156 |
| N Status (N0) | 0.403 (0.291-0.557) | | <0.001 | | 0.444 (0.294-0.669) | <0.001 |
| Resection margin (R0) | 0.568 (0.411-0.786) | | 0.001 | | 0.628 (0.411-0.959) | 0.031 |
| Histopathological Grading |  | |  | |  |  |
| G1 | Reference | | 0.051 | | Reference | 0.311 |
| G2 | 0.429 (0.183-1.005) | | 0.051 | | 0.473 (0.158-1.415) | 0.181 |
| G3 | 0.708 (0.500-1.000) | | 0.050 | | 0.963 (0.607-1.528) | 0.874 |
| Perineural sheath infiltration (Pn1) | 0.744 (0.363-1.525) | | 0.419 | |  |  |
| Lymphovascular invasion (L0) | 0.658 (0.464-0.931) | | 0.018 | | 1.234 (0.742-2.052) | 0.824 |
| Microvascular invasion (V0) | 0.503 (0.336-0.752) | | 0.001 | | 0.491 (0.299-0.807) | 0.005 |
| No adjuvant chemotherapy | 0.846 (0.541-1.324) | | 0.464 | |  |  |
| Carbohydrate Antigen 19-9 (U/ml) | 1.000 (1.000-1.000) | | 0.897 | |  |  |
|  |  | |  | |  |  |
| **N0 patients** | | | | | | |
|  | Univariate | | | Multivariate | | |
| Variable | HR (95% CI) | *P* value | | HR (95% CI) | | *P* value |
| Age | 1.019 (0.995-1.044) | 0.128 | |  | |  |
| Gender (male) | 1.350 (0.830-2.196) | 0.227 | |  | |  |
| Body Mass Index | 1.046 (0.979-1.118) | 0.184 | |  | |  |
| Bismuth-Corlette > II | 0.738 (0.336-1.620) | 0.449 | |  | |  |
| T Stage <3 | 0.608 (0.375-0.985) | 0.043 | | 0.728 (0.393-1.349) | | 0.313 |
| Resection margin (R0) | 0.493 (0.294-0.826) | 0.007 | | 0.504 (0.264-0.959) | | 0.037 |
| Histopathological Grading |  |  | |  | |  |
| G1 | Reference | 0.300 | |  | |  |
| G2 | 0.556 (0.162-1.903) | 0.350 | |  | |  |
| G3 | 0.663 (0.385-1.143) | 0.139 | |  | |  |
| Perineural sheath infiltration (Pn1) | 0.719 (0.258-2.005) | 0.529 | |  | |  |
| Lymphovascular invasion (L0) | 0.950 (0.512-1.762) | 0.871 | |  | |  |
| Microvascular invasion (V0) | 0.465 (0.242-0.894) | 0.022 | | 0.642 (0.309-1.333) | | 0.234 |
| No adjuvant chemotherapy | 0.708 (0.319-1.572) | 0.396 | |  | |  |
| Carbohydrate Antigen 19-9 (U/ml) | 1.000 (1.000-1.000) | 0.832 | |  | |  |
|  |  | |  | |  |  |
| **N+ patients** | | | | | | |
|  | Univariate | | | Multivariate | | |
| Variable | HR (95% CI) | *P* value | | HR (95% CI) | | *P* value |
| Age | 1.016 (0.994-1.039) | 0.148 | |  | |  |
| Gender (male) | 1.226 (0.794-1.894) | 0.358 | |  | |  |
| Body Mass Index | 0.969 (0.501-1.872) | 0.924 | |  | |  |
| Bismuth-Corlette > II | 1.179 (0.580-2.393) | 0.649 | |  | |  |
| T Stage <3 | 0.891 (0.584-1.361) | 0.594 | |  | |  |
| Resection margin (R0) | 0.898 (0.584-1.382) | 0.626 | |  | |  |
| Histopathological Grading |  |  | |  | |  |
| G1 | Reference | 0.132 | | Reference | | 0.105 |
| G2 | 0.309 (0.093-1.028) | 0.055 | | 0.214 (0.049-0.937) | | 0.041 |
| G3 | 1.043 (0.661-1.646 | 0.855 | | 1.025 (0.609-1.727) | | 0.925 |
| Perineural sheath infiltration (Pn1) | 1.392 (0.502-3.862) | 0.525 | |  | |  |
| Lymphovascular invasion (L0) | 0.914 (0.560-1.491) | 0.719 | |  | |  |
| Microvascular invasion (V0) | 0.635 (0.380-1.061) | 0.083 | | 0.398 (0.212-0.748) | | 0.127 |
| No adjuvant chemotherapy | 2.227 (1.284-3.863) | 0.004 | | 2.635 (1.413-4.917) | | 0.002 |
| Carbohydrate Antigen 19-9 (U/ml) | 1.000 (1.000-1.000) | 0.112 | |  | |  |
|  |  |  | |  | |  |
|  |  | |  | |  |  |
